# Migraine and type 2 diabetes; is there any association?

**DOI:** 10.1186/s40200-016-0241-y

**Published:** 2016-09-08

**Authors:** Fatemeh Sadat Haghighi, Masoud Rahmanian, Nasim Namiranian, Seyed Masoud Arzaghi, Farzane Dehghan, Fahime Chavoshzade, Fariba Sepehri

**Affiliations:** 1 Diabetes Research Center, Shahid Sadoughi University of Medical Sciences, Yazd, Iran; 2 Elderly Health Research Center, Endocrinology and Metabolism Research Institute, Tehran University of Medical Sciences, Tehran, Iran; 3 Yazd Diabetes Research Center, Talar-e-Honar Alley, Shahid Sadoughi Blvd, Yazd, Iran

**Keywords:** Migraine, Type 2 diabetes mellitus, Prevalence

## Abstract

**Background:**

Migraine headache prevalence and triggers in type2 diabetes mellitus (T2DM) were investigated in previous studies but the results are contradictory. Therefore, in this study we examined the prevalence of migraine headache in diabetic patients in comparison with non-diabetic persons and its predisposing factors in 2014.

**Methods:**

We enrolled 147 volunteer patients with T2DM and 150 healthy persons referred to the Yazd Diabetes Research Center and the Central Laboratory of Yazd, respectively, in 2014. The data collection instrument was a self-conducted checklist. The checklist contained demographic, anthropometric and clinical characteristics and migraine diagnostic questions according to International Classification of Headache Disorders Second Edition (ICHD-II) criteria. We compared prevalence of migraine between two groups, and also evaluated relationship between above characteristics and migraine prevalence in both groups.

**Results:**

The prevalence of migraine in participants of diabetic and non-diabetic was 27.9 and 26 %, respectively (*p*-value = .406). The prevalence of migraine headache among in diabetic persons was significantly correlated with family history of migraine, diabetes duration and hypoglycemia attacks. Also, the migraine prevalence was significant more prevalent in T2DM patients with duration 6–10 years (*p*-value = 0.031). The percentage of HbA1C, type of anti-diabetic medication, BMI value and age in diabetic patients did not show any significant association with migraine.

**Conclusion:**

Although we observed no significant differences in prevalence of migraine between patients with T2DM and non-diabetic age and sex adjusted persons But, the occurrence of hypoglycemia attacks and T2DM duration were related to migraine prevalence. Decreasing hypoglycemia among long-time T2DM patients probably can decline migraine headache in this group of patients.

## Background

Type2 diabetes mellitus (T2DM) influences all ages, and its prevalence is increasing worldwide [1]. This disease is correlated with an enhanced risk of several other chronic disorders [2] and causes multitude complications such as diabetic neuropathy, retinopathy and cardiovascular disorders. Among the complications related to diabetes mellitus, association between migraine and diabetes is controversial. Migraine is a neurovascular disorder with complex pathophysiology and recurrent headache attacks. Duration of migraine headache is 4 to 72 h and is usually accompanied by nausea, vomiting, or sensibility to sound, light, or motion [3, 4]. Migraine associated with an extensive range of subtypes, multiple co-morbidities and a changeable prognosis and has been better investigated than other type of headaches [5]. A World Health Organization (WHO) review of universal data detected migraine to be one of the most common health diseases worldwide, and the most prevalent reason of headache consultation in America, South-East Asia, Europe, and the Western Pacific [6]. The reason of migraine is unknown [7] and several researches have shown that genetics and environmental factors are significant pathophysiologic causes of migraine [8]. Since migraine is a complex disease and several factors are involved it, numerous studies have investigated to discover its predisposing factors. Hypoglycemia and fasting that is seen commonly in diabetic patients are among these factors. So, we compared the prevalence of migraine in diabetic and non-diabetic people and evaluated relationship between migraine and some factors including age, sex, hypoglycemia induced by anti-diabetic drug, glycosylated hemoglobin (HbA_1c_), family history, educational level and job in diabetic and non-diabetic individuals in Yazd Diabetes Research Center in 2014.

## Methods

An HbA1C of 6.5 % is recommended as the cut of point for diagnosing diabetes. In this study, was used Japanese toso device and HPLC (High Performance Liquid Chromatography) technique to measure HbA1C levels. This cross-sectional study was carried out in type 2 diabetic patients referred to Yazd Diabetes Research Center in 2014. Sample size was calculated with sample size calculation formula for limited population. The study sample was 147 diabetic patients that were randomly selected among 10000 diabetic patients between 30 and 45 years-old referred to Yazd Diabetes Research Center in 2014. One hundred and fifty age and sex matched non-diabetic people were randomly selected from the Central Laboratory of Yazd. The exclusion criteria were patients with hypertension [4]. The samples were recruited in 2014. The data collection instrument was a self-conducted checklist. The checklist contained demographic information (sex, age, employment, education, family history of migraine in first degree relatives, history of hypoglycemia, duration of T2DM and anti-diabetic medications), anthropometric measurement (Body Mass Index value), Laboratory findings (Fasting Plasma Glucose, Post prandial plasma glucose and HbA1c tests) and migraine diagnostic questions according to International Classification of Headache Disorders Second Edition (ICHD-II) criteria. The universally accepted international classification of headache disorders, first published in 1988, has now been replaced by a second edition ICDH-2. It is important to implement the new edition immediately since there are many important changes compared with the first edition. Application of the new edition is the only way to assure that the same diagnostic approach and appropriate treatment are provided for all headache patients. The ICHD-II criteria for diagnosis of migraine are; The duration of no-remedy or failed treated episodes ranges from 4 to 72 h. The headaches are determined with containing at least two subsequent pain characteristics: unilateral status, pulsating state, moderate or severe intensity, Inability to perform physical activity. The headache is accompanied by at least one of the following signs: nausea or vomiting, photophobia, and sensitivity to sound. Also, the patient must have a history of at least five prior attacks according to the diagnostic criteria [7]. Weight and height of participants were measured by researcher, and BMI was calculated by dividing weight in kilograms by height in square meters. The BMI values ≤20 kg/m^2^, 20–25 kg/m^2^, ≥25 kg/m^2^ and ≥30 kg/m^2^, respectively, defined as underweight, normal weight, overweight and obese [9]. The sample size was calculated by comparison of two prevalences. The power of study was 85 %, α was 0.05 and the differences between groups was 0.2.

The data obtained using descriptive statistics and inferential statistics (chi-square test) and analysis was done using SPSS software 20. *P*-value of < .05 was considered as statistically significant.

This study is adopted in Ethics Committee of Yazd Diabetes Research Center, based on the Helsinki Declaration.

## Results

**Fig. 1:**
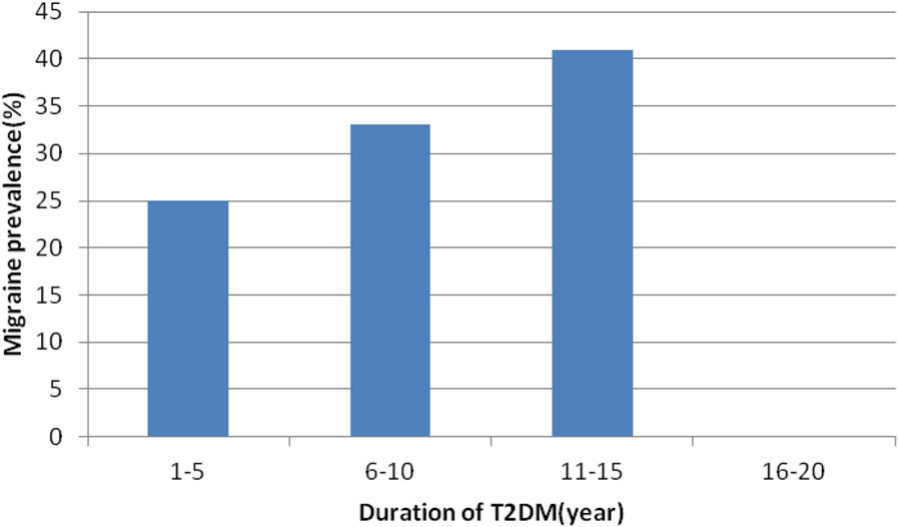
Migraine prevalence with *p*-value of 0.031 related to T2DM duration

**Fig. 2:**
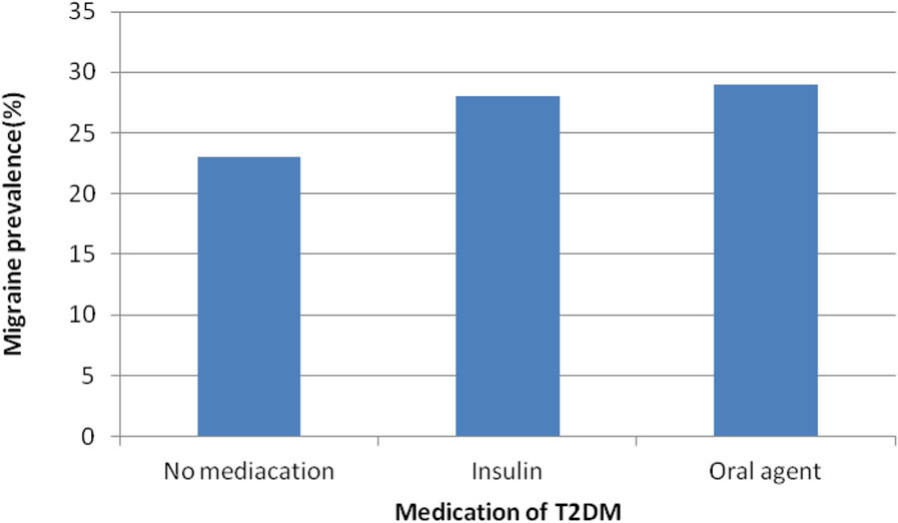
The prevalence of migraine between diabetic patients with diverse medications of T2DM (*p*-value of .549)

**Fig. 3:**
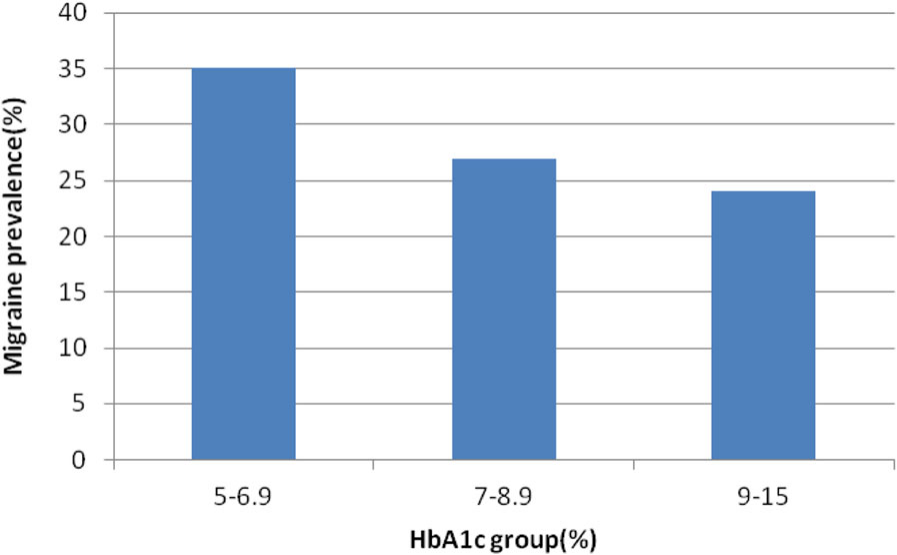
The prevalence of migraine between diabetic patients with diverse levels of HbA1c (*p*-value of .306)

**Fig. 4:**
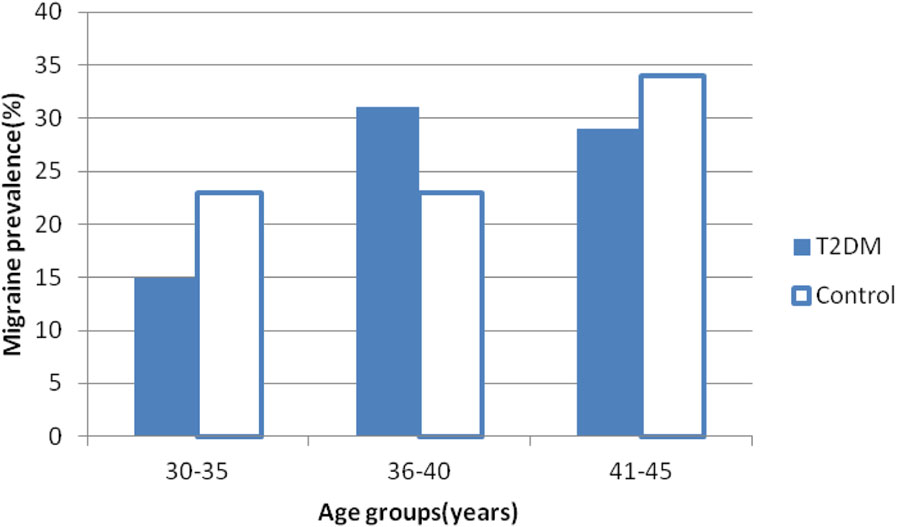
The age groups had no significant difference in the prevalence of migraine in both two group of diabetics and non-diabetics (*p*-values of .471 and .251, respectively)

**Fig. 5:**
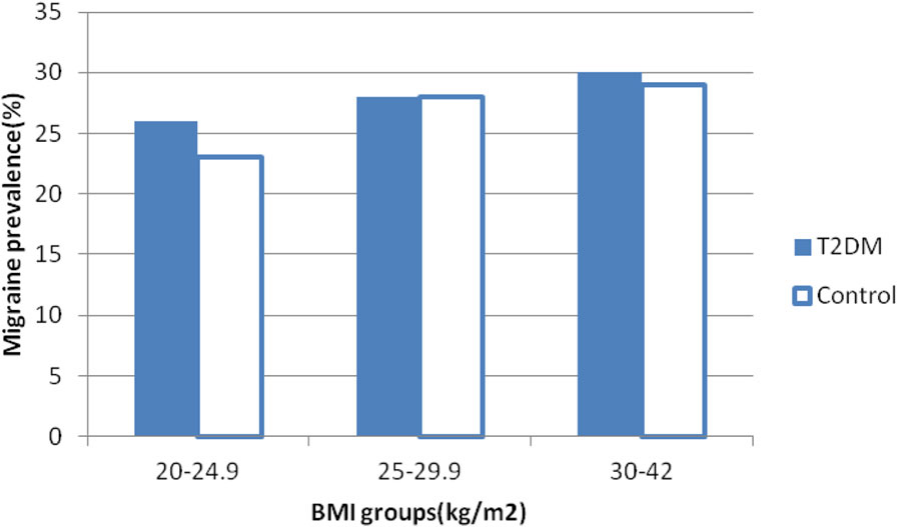
The BMI groups had no significant difference in the prevalence of migraine in both two group of diabetics and non-diabetics (*p*-values of .712 and .492, respectively

The prevalence of migraine in diabetics and non-diabetics were 27.9 and 26 %, respectively (*P*-value = .406) (Table 1) . The prevalence of migraine in diabetic patients with a family history of migraine in first degree relatives and without the history was 50 and 20.9 %, respectively (*p*-value > 0.001), and the prevalence of migraine in diabetic persons with a history of hypoglycemia and without the history was 41.2 and 16.7 %, respectively (*p*-value > 0.001). Duration of diabetes was significantly associated with migraine prevalence (*p*-value = .031) (Fig. 1). The migraine prevalence in patients treated with insulin, oral anti-diabetic agents and no medication were 27.9, 29.5 and 23.1 %, respectively (*p*-value = 0.549) (Fig. 2). Also, no significant difference was found in the prevalence of migraine in diabetic patients with high levels of HbA_1c_ (poor control of diabetics) (Fig. 3). Also, the age and BMI in diabetics and non-diabetics indicated no significant correlation with migraine prevalence (Figs. 4 and 5). The complementary comparisons between two groups were described in Table 2.

**Table 1:** Background characteristic of participants without and with Type 2 diabetes mellitus

Variable		Diabetic group, *n* = 147	Non-diabetic group, *n* = 150
		No. (%)	No. (%)
Sex	Male	55 (37.4)	48 (32)
	Female	92 (62.6)	102 (68)
Employment	No	87 (59.2)	84 (56)
	Yes	60 (40.8)	66 (44)
Education	Below diploma	94 (64.8)	52 (34.7)
	Diploma and upper	51 (35.2)	98 (65.3)
History of migraine in first degree relatives	No	110 (75.3)	109 (72.7)
	Yes	36 (24.7)	41 (27.3)
Age (years)	30–35	13 (8.8)	61 (40.7)
	36–40	26 (17.7)	44 (29.3)
	41–45	108 (73.5)	45 (30)
BMI (kg/m^2^)	20–25	35 (24)	53 (35.3)
	25–30	67 (45.9)	66 (44)
	30–42	44 (30.1)	31 (20.7)
Medication	No medication	26 (17.7)	……
	Insulin	43 (29.3)	……
	Oral agants	78 (53)	……
Hypoglycemia	No	78 (53.4)	……
	Yes	68 (46.6)	……
HbA1c (%)	5–7	31 (21)	……
	7–9	63 (42.9)	……
	9–15	53 (36.1)	……
Duration of diabetes (years)	1–5	85 (57.8)	……
	6–10	30 (20.4)	……
	11–15	22 (15)	……
	16–20	10 (6.8)	……
Migraine	No	106 (72.1)	111 (74)
	Yes	41 (27.9)	39 (26)

*BMI* indicates body mass index, *HbA1c (%)*, percentage of blood glycosylated hemoglobin, *No. (%)* frequency of variables

**Table 2:** Association between migraine prevalence and demographic, anthropometric, and clinical characteristics in patients with type 2 diabetes and healthy people

Variable		Diabetic group			Non diabetic group			
		n	%	*p*-value	n	%	*p*-value	*p*-value*
Sex	Male	4	7.3	<.001	4	8.3	<.001	<.001
	Female	37	40.2		35	34.3		
Employment	No	34	39.1	<.001	29	34.5	.006	<.001
	Yes	7	11.7		10	15.2		
Education	Below diploma	32	34	.013	20	38.5	.010	.001
	Diploma and upper	8	15.7		19	19.4		
History of migraine in first degree relatives	No	23	20.9	.001	17	15.6	<.001	<.001
	Yes	18	50		22	53.7		
Age (years)	30–35	2	15.4		14	23		
	36–40	8	30.8	.471	10	22.7	.251	.172
	41–45	31	28.7		15	33.3		
BMI (kg/m^2^)	20–25	9	25.7		12	22.6		
	25–30	19	28.4	.712	18	27.3	.492	.426
	30–42	13	29.5		9	29		
Medication	No drug	6	23.1		……	……	……	……
	Insulin	12	27.9	.549	……	……	……	……
	Oral agants	23	29.5		……	……	……	……
Hypoglycemia	No	13	16.7	.001	……	……	……	……
	Yes	28	41.2					
HbA1c (%)	5–7	11	35.5		……	……	……	……
	7–9	17	27	.306	……	……	……	……
	9–15	13	24.5		……	……	……	……
Duration of diabetes (years)	1–5	22	25.9		……	……	……	……
	6–10	10	33.3	.031	……	……	……	……
	11–15	9	40.9		……	……	……	……
	16–20	0			……	……	……	……

BMI indicates body mass index; HbA1c (%), percentage of blood glycosylated hemoglobin; n, the number of persons with migraine; %, frequency of migraine, *p*-value*, *p*-value between two groups of diabetics and non-diabetics

## Discussion

We observed no significant difference in the prevalence of migraine between patients with diabetes and non-diabetic age and sex matched group. Also, among factors that we studied in diabetic patients, only the family history of migraine in first degree relatives, the history of hypoglycemia and duration of T2DM were significantly related to migraine prevalence.

The correlation between migraine and diabetes is yet unknown, and there are conflicting results about prevalence of migraine in persons with diabetes [10]. In our study, the prevalence of migraine in diabetic and non-diabetic participants was 27.9 and 26 %, respectively, and the difference of migraine prevalence between two groups was statistically non significance (*p*-value = .406). Several previous studies have been designed to investigate the relation between migraine and diabetes [11, 12]. Some studies illustrated lower prevalence of migraine in patients with diabetes [13, 14], a similar [15] or higher prevalence [12, 16] was detected in other researches. The cohort study in 2012, found no correlation between migraine and T2DM [13]. A study suggested that the blood glucose level is the key and special triggering mechanism of migraine in some persons; Blau et al. selected 36 patients with both migraine and T2DM. Five patients lost their migraine perfectly or had a considerable decrease in their attacks with the onset or control of T2DM, and decline frequency and intensity of migraine in remarkable number of patients were found after developing T2DM; but, in 21 patients, diabetes in no way impressed migraine [17]. In addition, insulin resistance that is the core pathophysiology of T2DM has been correlated in some patients with migraine [18]. For example, a recent study indicated that insulin sensitivity is impaired in patients with migraine [5, 19], and another research confirmed an important relation between insulin resistance and migraine in sixty non-obese patients with migraine [20].

In our study, there was no significant difference in migraine prevalence between the age groups of 30–35, 36–40 and 41–45 years old in both two groups of diabetics and non-diabetics (*p*-values of .471 and .251, respectively). The previous studies indicated that highest prevalence of migraine occurs between ages 22 to 55 years old [21]. Berge LI et al. represented a considerable decreased prevalence of migraine among older patients with T2DM. This preventive effect of diabetes on migraine was only found among Patients older than 50 years old. But, prevalence of migraine in patients 40 to 49 years old was identical with the non-diabetic group and in patients less than 40 years old was higher. These findings suggested the presence of a potent age-related factor in migraine occurrence [2].

In our study, the prevalence of migraine in persons with T2DM duration of 1–5, 6–10, 11–15 years was 25.9, 33.3 and 40.9 %, respectively, and migraine prevalence with *p*-value of 0.031 related to T2DM duration. There was no case of migraine in T2DM duration of 16–20 years. Aamodt et al. represented a reverse correlation between Duration of T2DM and migraine prevalence in middle-aged and elderly patients for more than 13 years. As a result of this study was conjectured that diabetes mellitus may in some paths protect versus migraine, or vice versa. The cause of relationship between migraine prevalence and diabetes duration is unknown, but might be correlated to pathophysiological abnormalities [11]. One of these mechanisms may be a reduction of some neurotransmitters (e.g. nitric oxide, noradrenalin, and substance P) in nerve terminals of patients with diabetic neuropathy that this may be associated to migraine pathophysiology [20].

In our study, the prevalence of migraine in patients with diabetes with a history of hypoglycemia was significantly higher than patients without a history of hypoglycemia (*P*-value of .001). Hypoglycemia may accelerate headache in some patients with T2DM (and non-diabetic persons) [22] and is one of the side effects of anti-diabetic medication. The brain function depends on a continuous glucose supply and is vulnerable to any deficiency of glucose. So, the brain is one of the first organs that affected by reduced blood glucose levels [23]. In a study, overnight hypoglycemia accelerated migraine headaches in some patients with both migraine and T2DM. It expresses the relationship between blood glucose levels and the occurrence of migraine headaches [17].

Migraine happens more prevalently in adult women than men [24]. In the United States, one-year prevalence of migraine is estimated 18 % in women, which is three times more prevalent in men [25]. Also, a study in Turkey showed the lifetime prevalence of migraine in men and women as 7.9 % and 17.1 % [26]. The results of our research confirm the findings of previous studies; a higher prevalence of migraine in both women of diabetics and non-diabetics groups (*p*-value of < .001).

In previous studies it has been suggested that migraine issued from a genetic basis. People with a family history of migraine are more susceptible to migraine. The results of our study about two groups of diabetics and control confirmed previous findings. Migraine is a multifactorial disease and has a complex inheritance [4]. Globally, migraine prevalence is highest in the Americas and Europe and lowest in Africa and Asia . Also, a resembling race pattern is observed in the United States, where number of whites is more than African- and Asian-Americans, proposing race-related diversities in genetic vulnerability to migraine [3].

Some medicines used to treat metabolic disorders indicated an important effect in prophylaxis of migraine [18]. For example, in a study, patients using anti-diabetic medications had an overall decreased prevalence of medically treated migraine compared with the non-diabetic persons. Although young patients taking oral anti-diabetic agents had an elevated prevalence of medically treated migraine, the prevalence decreased with increasing age to about the identical reduced prevalence for all kinds of T2DM treatment in Patients 60–90 years old [2]. In our study, type of medication of diabetes was classified to three categories; patients without medication, patients using insulin, and patients treated with oral anti-diabetic agents. The prevalence of migraine in these three medication groups were 23.1, 27.9, and 29.5 %, respectively (*p*-value = .549).

The correlation between migraine and obesity remains unclear, and results of various studies are inconsistent [21]. In two population-based studies, BMI was not correlated with the episodic migraine prevalence [27, 28]. However, two other studies indicated a positive relation between migraine and elevated BMI [29, 30]. Also, some researchers conclude that patients with underweight are more likely to have an elevated risk for migraine [29, 31]. In our study, no significant association was observed between migraine and the levels of BMI in both groups.

Our study showed that the HbA_1c_ level is not associated with prevalence of migraine in diabetic patients. A study on patients with both migraine and T2DM showed that 5 patients of 36 participants lost their migraine entirely or had a considerable decline in their attacks with the onset or control of T2DM [17]. But, in other studies on patients with migraine, no important correlations were shown between HbA_1c_ level and prevalence of migraine [5].

The cause of controversy in results regarding the association between studied cases and migraine prevalence are not completely explicit, but several methodological diversities may underlie this difference. For example, some of the studies used measured height and weight [29, 30, 32] and others recorded weight and height as self-reported [27], [28, 33, 34]. There have also been various methods used to diagnose migraine, so creating problem in interpreting and comparing results across studies.

## Conclusion

We observed no significant difference in the prevalence of migraine in patients with diabetes and non-diabetic persons. Also, among factors that were studied in diabetic patients, positive family history of migraine in first degree relatives, history of hypoglycemia and T2DM duration were significantly correlated with migraine prevalence. Decreasing hypoglycemia among long-time T2DM patients probably can decline migraine headache in this group of patients.

## Abbreviations

T2DM, Type2 diabetes mellitus; ICHD-II, international classification of headache disorders second edition; HbA1C, Glycated hemoglobin; BMI, body mass index; WHO, World Health Organization; HPLC, high performance liquid chromatography; SPSS, statistical package for the social sciences.
